# Alum Treatment of Whooping Cough

**Published:** 1884-12

**Authors:** 


### ALUM TREATMENT OF WHOOPING COUGH.

NOT many years ago alum was one of the favorite remedies for the relief of pertussis, but of late it has been almost entirely superseded by other less unpalatable drugs. Now it seems to be again entering upon a time of favor and appreciation. Dr. Warfvine, of Stockholm, records a series of cases of pertussis of varying degrees of severity in which he exhibited the remedy, as a rule, as soon as the characteristic symptoms were declared. The earlier the treatment was begun the better were the results obtained. In one case of a boy, eight years of age, who had had a cough for three weeks, and who had just begun to whoop, the symptoms disappeared entirely after the use of alum, in a two per cent. solution, for two weeks. In another case of a girl, six years of age, who had from twenty to twenty-five moderately severe attacks in a day, the cough was cured in ten days by the same means. The remedy was given usually in a one or two per cent. solution, in equal parts of water and orange syrup, in the dose of a teaspoonful four times a day. Even in the later stages of the disease, the attacks seemed to be greatly reduced in frequency and severity when alum was exhibited to the exclusion of all other remedies.

**A Simple Remedy in Diarrhœa.**—In the late summer and autumn, when fruit is so abundant, any simple remedy for diarrhœa, and what is familiarly called "bowel complaint," is worth knowing and remembering. Such a one was recommended by Dr. T. E. Stellwagen several years ago, in his edition of Coleman's "Dental Surgery,' and more recently in the pages of one of the journals. It is simply *vinegar*, preferably sound cider vinegar. The dose is about two ounces for an adult, and should be swallowed "neat," without admixture with water.

It may also be given to infants with excellent results. To a babe one year old a teaspoonful of moderately diluted vinegar would be the proper dose.

Its effect is to check pain, tenesmus, and tormina at once, to relieve the chills and cramps when present, and to disseminate a feeling of warmth and comfort over the body.

Even in cases of chronic diarrhœa which have long resisted treatment, this househould remedy has succeeded in checking the discharges and correcting the sub-inflammatory condition of the membranes.

That state of life is the most happy where superfluities are not required, and where necessaries are not wanting.

